# Supplementary material for: Prediction of Glomerular Filtration Rate Following Partial Nephrectomy for Localized Renal Cell Carcinoma with Different Machine Learning Techniques
Source: Cancers (Basel). 2025 May 13;17(10):1647. doi: 10.3390/cancers17101647 (PMC12110722; doi:10.3390/cancers17101647)

Supplementary Figure S1. Selection flowchart.

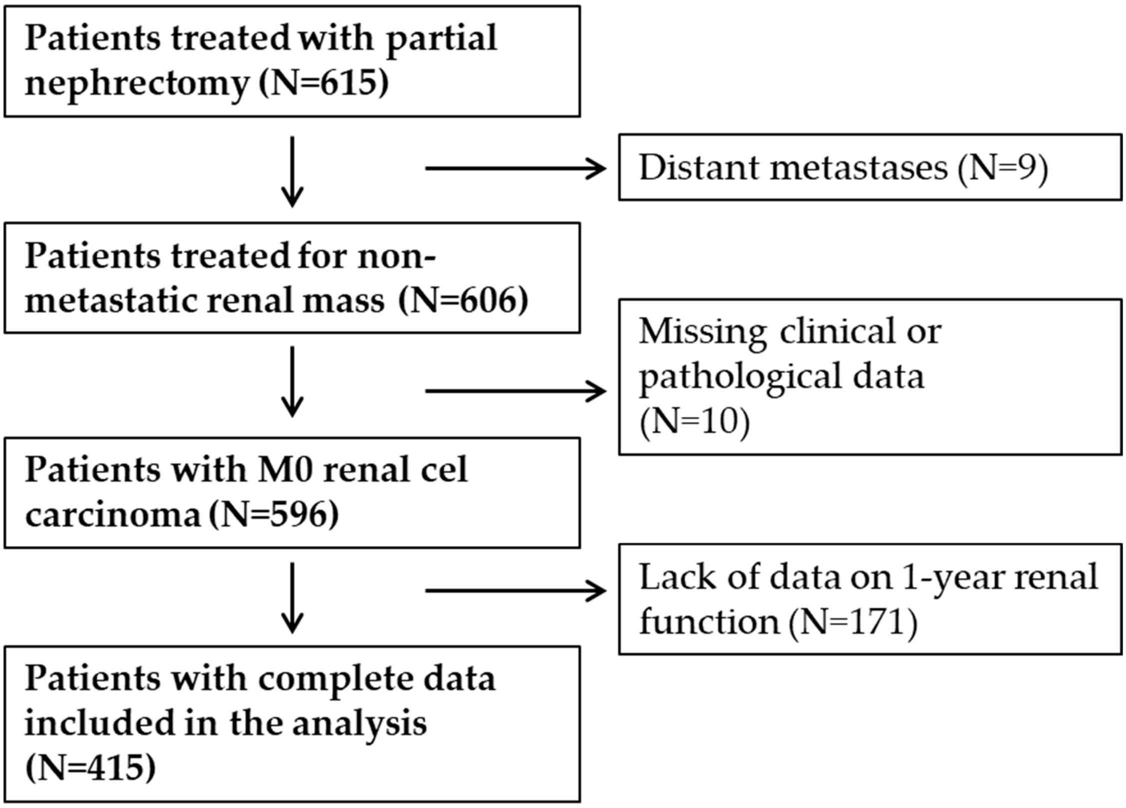

Supplementary Figure S2. Correlation matrix of preoperative clinical variables using the Spearman test.

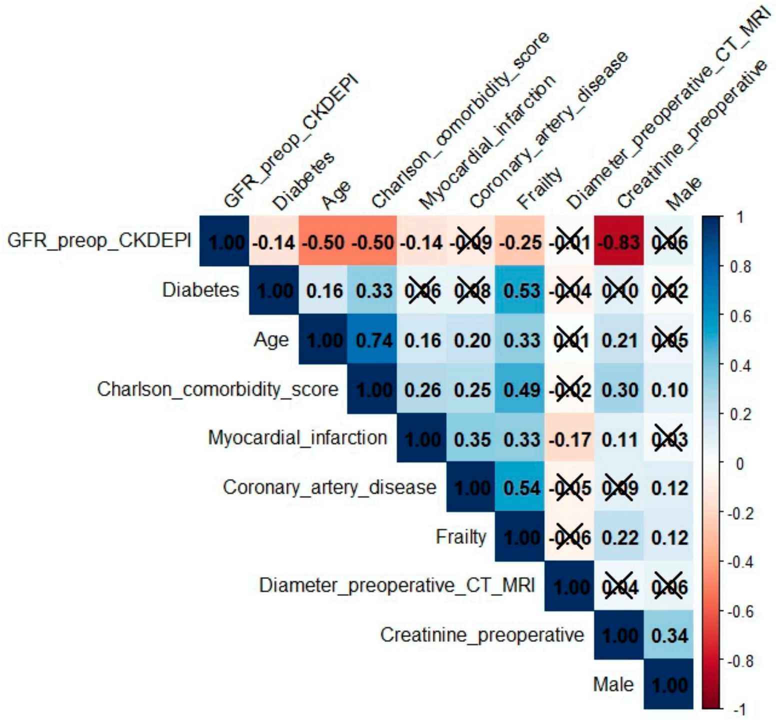

X – indicates lack of statistical significance (p > 0.05)

Supplementary Figure S3. A histogram and quantile-quantile plot illustrating the distribution of residuals in the linear regression model (A). A plot presenting the values of residuals versus fitted values assessing homoscedasticity (B).

A

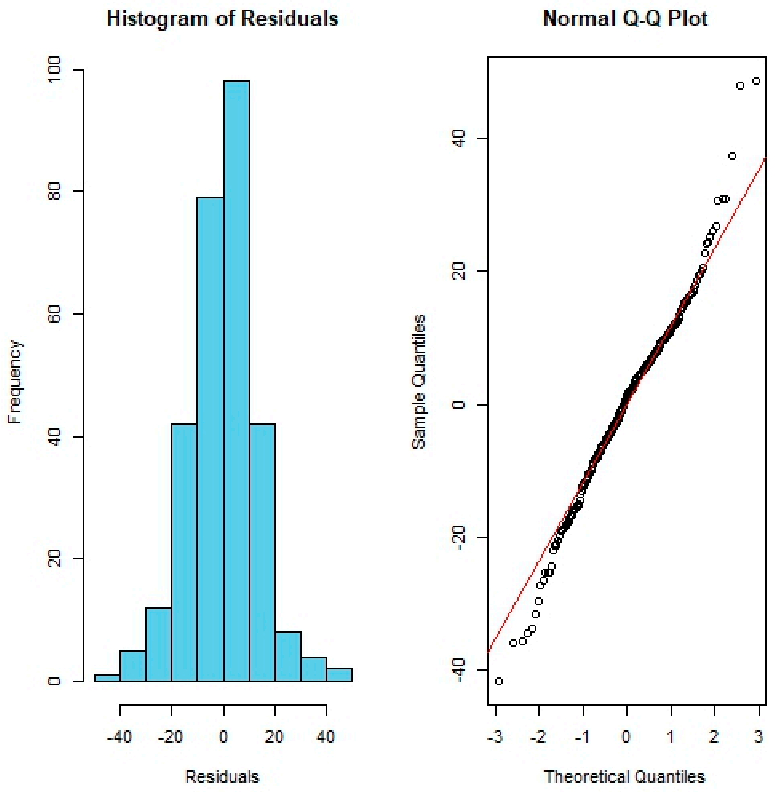

B

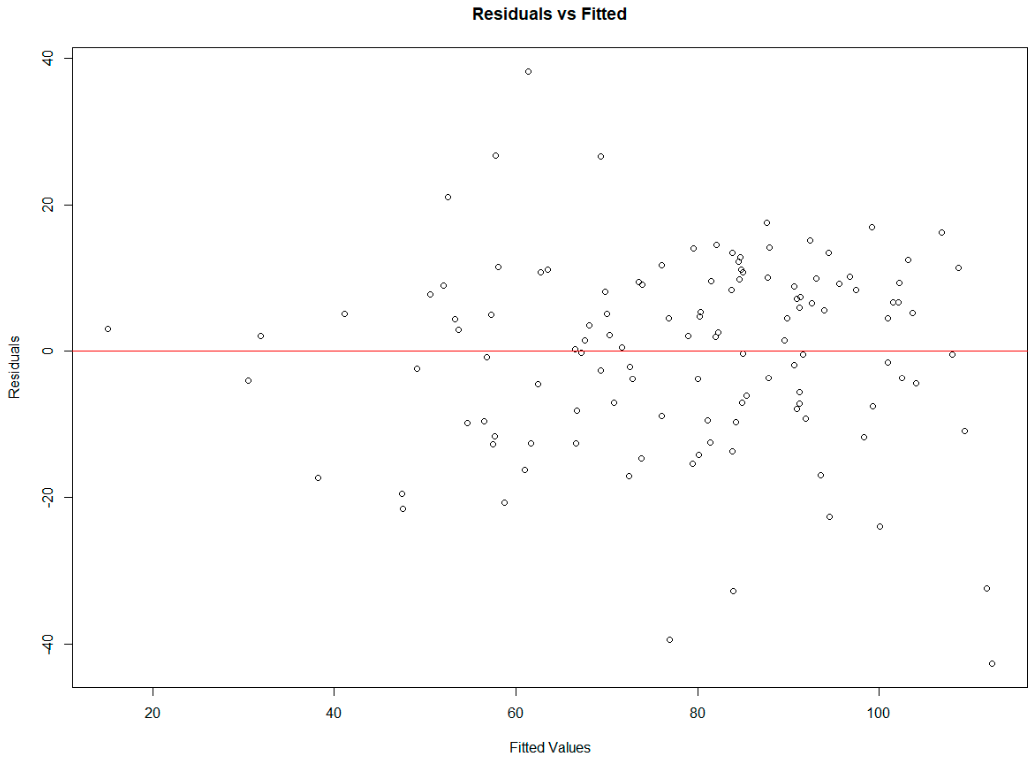

Supplement: Supplementary file 1 [file cancers-17-01647-s001.zip › cancers-3564938-supplementary.pdf]
